# Prospective Evaluation of Health Care Provider and Patient Assessments in Chemotherapy-Induced Peripheral Neurotoxicity

**DOI:** 10.1212/WNL.0000000000012300

**Published:** 2021-08-17

**Authors:** Paola Alberti, Davide P. Bernasconi, David R. Cornblath, Ingemar S.J. Merkies, Susanna B. Park, Roser Velasco, Jordi Bruna, Dimitri Psimaras, Susanne Koeppen, Andrea Pace, Susan G. Dorsey, Andreas A. Argyriou, Haralabos P. Kalofonos, Chiara Briani, Angelo Schenone, Catharina G. Faber, Anna Mazzeo, Wolfgang Grisold, MariaGrazia Valsecchi, Guido Cavaletti, on behalf of the CI-PeriNomS group

**Affiliations:** From Experimental Neurology Unit (P.A., G.C.) and Bicocca Bioinformatics Biostatistics and Bioimaging Centre–B4 (D.P.B., M.G.V.), School of Medicine and Surgery, University of Milano-Bicocca, Monza; NeuroMI (Milan Center for Neuroscience) (P.A., G.C.), Milan, Italy; Johns Hopkins University School of Medicine (D.R.C.), Baltimore, MD; Department of Neurology (I.S.J.M., C.G.F.), Maastricht University Medical Centre, the Netherlands; Department of Neurology (I.S.J.M.), St Elisabeth Hospital, Willemstad, Curaçao; University of New South Wales (S.B.P.), Sydney, Australia; Unit of Neuro-Oncology, Neurology Department (R.V., J.B.), Hospital Universitari de Bellvitge-ICO l’Hospitalet, IDIBELL, L'Hospitalet de Llobregat, Barcelona; Institute of Neurosciences and Department of Cell Biology, Physiology and Immunology (R.V., J.B.), Universitat Autònoma de Barcelona, Centro de Investigación Biomédica en Red sobre Enfermedades Neurodegenerativas (CIBERNED), Bellaterra, Spain; Service de Neurologie Mazarin (D.P.), Hôpital de la Pitié-Salpêtrière, Université Paris Sorbonne, Paris, France; Department of Neurology and West German Cancer Center (S.K.), University of Essen, Germany; IRCCS Regina Elena Cancer Institute (A.P.), Neuro-Oncology Unit, Rome, Italy; Department of Pain & Translational Symptom Science (S.G.D.), University of Maryland Baltimore; Neurological Department (A.A.A.), Saint Andrew's General Hospital of Patras; Department of Medicine, Division of Oncology (A.A.A., H.P.K.), Medical School, University of Patras, Greece; Department of Neurosciences (C.B.), University of Padova; Department of Neurosciences, Rehabilitation, Ophthalmology, Genetic and Maternal and Infantile Sciences (DINOGMI) (A.S.), University of Genova; Unit of Neurology and Neuromuscular Diseases (A.M.), Department of Clinical and Experimental Medicine, University of Messina, Italy; and Ludwig Boltzmann Institute for Experimental und Clinical Traumatology (W.G.), Vienna, Austria.

## Abstract

**Background and Objective:**

There is no agreement on the gold standard for detection and grading of chemotherapy-induced peripheral neurotoxicity (CIPN) in clinical trials. The objective is to perform an observational prospective study to assess and compare patient-based and physician-based methods for detection and grading of CIPN.

**Methods:**

Consecutive patients, aged 18 years or older, candidates for neurotoxic chemotherapy, were enrolled in the United States, European Union, or Australia. A trained investigator performed physician-based scales (Total Neuropathy Score–clinical [TNSc], used to calculate Total Neuropathy Score–nurse [TNSn]) and supervised the patient-completed questionnaire (Functional Assessment of Cancer Treatment/Gynecologic Oncology Group–Neurotoxicity [FACT/GOG-NTX]). Evaluations were performed before and at the end of chemotherapy. On participants without neuropathy at baseline, we assessed the association between TNSc, TNSn, and FACT/GOG-NTX. Considering a previously established minimal clinically important difference (MCID) for FACT/GOG-NTX, we identified participants with and without a clinically important deterioration according to this scale. Then, we calculated the MCID for TNSc and TNSn as the difference in the mean change score of these scales between the 2 groups.

**Results:**

Data from 254 participants were available: 180 (71%) had normal neurologic status at baseline. At the end of the study, 88% of participants developed any grade of neuropathy. TNSc, TNSn, and FACT/GOG-NTX showed good responsiveness (standardized mean change from baseline to end of chemotherapy >1 for all scales). On the 153 participants without neuropathy at baseline and treated with a known neurotoxic chemotherapy regimen, we verified a moderate correlation in both TNSc and TNSn scores with FACT/GOG-NTX (Spearman correlation index *r* = 0.6). On the same sample, considering as clinically important a change in the FACT/GOG-NTX score of at least 3.3 points, the MCID was 3.7 for TNSc and 2.8 for the TNSn.

**Conclusions:**

MCID for TNSc and TNSn were calculated and the TNSn can be considered a reliable alternative objective clinical assessment if a more extended neurologic examination is not possible. The FACT/GOG-NTX score can be reduced to 7 items and these items correlate well with the TNSc and TNSn.

**Classification of Evidence:**

This study provides Class III evidence that a patient-completed questionnaire and nurse-assessed scale correlate with a physician-assessed scale.

Chemotherapy-induced peripheral neurotoxicity (CIPN) from widely used anticancer drugs is a major issue in oncology daily practice.^1-3^ CIPN has a significant effect on participants both during^4^ and after antineoplastic treatment.^5-12^ Prevention or treatment of CIPN are important unmet clinical needs.^13^ A major reason for the lack of effective treatments is the incomplete knowledge of CIPN pathogenesis.^6-9,14^ However, another issue in clinical trials is the lack of a gold standard for CIPN detection and grading,^15^ leading to multiple and different rating instruments. To fill these gaps, we performed a longitudinal study on a real-life population of participants with cancer from baseline (i.e., before chemotherapy administration) to treatment completion. Based on several previous methodologic studies,^16-24^ a combination of clinician-reported outcome (CRO) as well as patient-reported outcome (PRO) measures seems to be the most reliable approach. Based on these results, our aim was to address several questions about currently used assessment tools: Are the National Cancer Institute Common Terminology Criteria for Adverse Events (NCI-CTCAE), the Total Neuropathy Score clinical version (TNSc) and its novel nurse-assessed version (TNSn), and the Functional Assessment of Cancer Treatment/Gynecologic Oncology Group–Neurotoxicity (FACT/GOG-NTX) scales responsive to the occurrence of CIPN in this population? How do the TNSc and TNSn compare? What are the correlations among the variation from baseline to end of treatment of TNSc, TNSn, and FACT/GOG-NTX in a population of participants receiving anticancer drugs? Are there shorter versions of FACT/GOG-NTX that might be as valuable as the complete version? What is the minimal clinically important difference (MCID) for the TNSc and TNSn?

## Methods

### Study Design

This is an international, multicenter (14 sites) trial involving European, American, and Australian centers primarily aimed at definition of the MCID for TNSc and TNSn and at the assessment of the possibility to used reduced FACT/GOG-NTX versions at the same level of reliability of the full version.

### Standard Protocol Approvals, Registrations, and Patient Consents

Adult participants were enrolled at each participating center after approval from local institutional review boards/ethics committees and written informed consent was obtained from each participant before entering the study.

### Study Design

Consecutive participants were age 18 years or older and candidates for neurotoxic chemotherapy for colorectal, breast, or lung cancers with noninvestigational drugs. Participants with potential confounding factors for CIPN were excluded (i.e., brain metastases, peripheral nerve damage due to other cause). At each center, a specifically trained investigator performed the selected health care provider–assessed scales, NCI-CTCAE (items “peripheral neuropathy–motor” and “peripheral neuropathy–sensory” of NCI-CTCAE v4.0 were used) and TNSc, and supervised the patient-completed questionnaire, FACT/GOG-NTX (version 4, items NTX1-9 and item HI12 and item An6), at baseline (before first chemotherapy cycle, T0) and at the end of all chemotherapy cycles (T1). Participants were evaluated before chemotherapy initiation and at its completion. Demographic and medical history were recorded. As TNSn is calculated from 5 of the 7 items of the TNSc, the TNSn was calculated for each participant at each visit where the TNSc was obtained. eTable 1 (available at Bicocca Open Archive Research Database [BOARD],^25^ board.unimib.it/research-data/) provides a detailed description of TNSc and TNSn items and eTable 2 (available at BOARD^25^) FACT-GOG-NTX items.

We first assessed the internal responsiveness of NCI-CTCAE, TNSc, TNSn, and FACT/GOG-NTX on the complete sample including participants with neuropathy at entry. We then used the sample of participants without neuropathy at entry (i.e., TNSc score 0 at baseline) to compare TNSc and TNSn; to assess correlations among TNSc, TNSn, and FACT/GOG-NTX in a population of participants receiving platinum, taxanes, or a combination of the 2 drugs; to assess whether shorter versions of FACT/GOG-NTX might provide the same information as the complete version; and to calculate the MCID for TNSc and TNSn.

### Statistical Analysis

Characteristics of the participants were summarized using numbers and percentages for categorical variables and mean with SD for continuous variables. The flow chart depicted in eFigure 1 (available at BOARD,^25^ board.unimib.it/research-data/) describes the size of the subsample of participants used in each analysis.

The responsiveness of TNSc, TNSn, and FACT/GOG-NTX scales was assessed by estimating several measures of the effect size of the score change between baseline and the end of treatment (table 1). The analysis of the internal responsiveness of NCI-CTCAE, TNSc, TNSn, and FACT/GOG-NTX was performed using all the available information, i.e., for each scale, data of participants with nonmissing values on every item at all visits were used. For NCI-CTCAE, a binomial test comparing the proportion of worsened participants according to each item was applied. For the other scales, a paired *t* test was performed and the effect size measures described in Husted et al.^26^ were estimated together with a 95% confidence interval (CI). All these measures consist of a ratio between the mean score change from T0 to T1 and an estimate of the score variability.

**Table 1:**
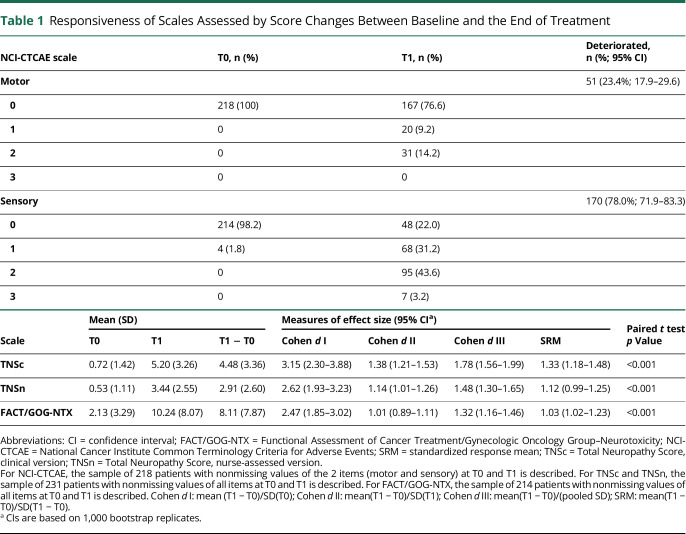
Responsiveness of Scales Assessed by Score Changes Between Baseline and the End of Treatment

The following analyses were performed on the 153 participants treated with a specified neurotoxic chemotherapy regimen, with a normal neurologic status at baseline, and with nonmissing items of TNSc, TNSn, and FACT/GOG-NTX at every time point. We compared the TNSc and the TNSn at T1 both graphically and using the Spearman correlation index. Differences in neurologic deterioration at the end of the follow-up according to TNSc and TNSn (categorization was based on a TNSc severity group subdivision,^27^ as follows: score 0, score 1–8, score 9–16, higher than 16; however, the highest score in our population was 15, therefore, we had 3 groups: 0, 1–8, and 9–15 according to Total Neuropathy Score [TNS]) between chemotherapy regimens were assessed using Fisher test. The association between deterioration according to FACT/GOG-NTX and TNSc or TNSn groups at T1 was checked using Kruskal-Wallis test and drawing boxplots. This analysis was repeated after stratifying by chemotherapy regimen. We then assessed whether shorter versions of FACT/GOG-NTX might provide the same information as the complete version. This was done by checking the association between deterioration of each single FACT/GOG-NTX item and TNSc or TNSn groups at T1, using the χ^2^ test for trend. Lastly, an anchor-based approach was applied to assess the MCID for TNSc and TNSn scale. This approach is recommended over distribution-based approaches (focusing purely on a “statistically relevant” change) when at least 1 external indicator of the smallest clinically meaningful change, serving as the anchor, is available.^28^ The idea consists of defining a group of participants with a relevant change based on the anchor measure and then comparing values of the scale of interest in this group with the group of participants where no change was observed. The direction of change (i.e., participants getting worse or getting better) should be taken into account. We relied on a previously established MCID for FACT/GOG-NTX to identify participants with and without a clinically important deterioration according to this scale. Then, we calculated the MCID for TNSc and TNSn as the difference in the mean change score of these scales between the 2 groups.

All analysis was carried out using R statistical package (version 3.6.0).

### Data Availability

Data will be made available upon request to the corresponding author.

## Results

### Description of the Study Population

Among the whole sample of 254 participants, about 50% had breast cancer (eTable 3, available at BOARD,^25^ board.unimib.it/research-data/). About 80% of participants were women with a mean age of ≈56 years. Colorectal cancer made up the next largest group, with about 22% in each population. About 50% of participants received a taxane alone, ≈34% received a platinum-containing agent, and just under 20% received both.

### Analysis of the Internal Responsiveness of NCI-CTCAE, TNSc, TNSn, and FACT/GOG-NTX Based on the Whole Sample of Participants With Nonmissing Values of the Scales at T0 and T1

As an initial analysis, we evaluated on participants of the whole population with completely measured scales at T0 and T1 the internal responsiveness of NCI-CTCAE (218 participants), TNSc (231), TNSn (231), and FACT/GOG-NTX (214) scales selected as study outcome measures. A description of the overall population and of the populations analyzed for each scale is provided in eTable 3 and eFigure 1 (available at BOARD,^25^ board.unimib.it/research-data/). Concerning the responsiveness of NCI-CTCAE, the percentage of participants with an increased score was 23.4% and 78.0% for motor and sensory items, respectively (both significantly higher than 0) (table 1). For all scales, the final score consistently increased on average by more than 1 SD, regardless of which type of SD is considered in the calculation (SD of the score at T0, SD at T1, an average of the previous 2, or SD of the change T1–T0). In other words, all effect sizes were greater than 1 and all the lower bounds of the corresponding 95% CIs were above 0.8, which is commonly considered as a threshold for large responsiveness.^26^

### Descriptive Statistics of the Selected Study Population (No Neuropathy at T0, No Missing Data on TNSc, and FACT/GOG-NTX, Treated With a Known Neurotoxic Regimen)

When stratifying for the neurologic status at study entry, 171 participants out of the original cohort of 254 had normal neurologic status at study entry and among these 155 had a complete FACT/GOG-NTX score at each time point. Among these, for 2 participants, information about the chemotherapy regimen received was missing. Thus, we excluded these 2 participants and analyzed the final sample of 153 participants. eTable 4 (available at BOARD^25^) gives a general overview of the study population, overall and stratified by TNSc category at T1. Breast cancer and colorectal cancer were most prevalent; therefore, the regimens administered contained taxanes, platinum compounds, or a combination of both classes.

### Neuropathy Course Over the Observational Period and Comparison Between TNSc and TNSn at T1

**Figure 1:**
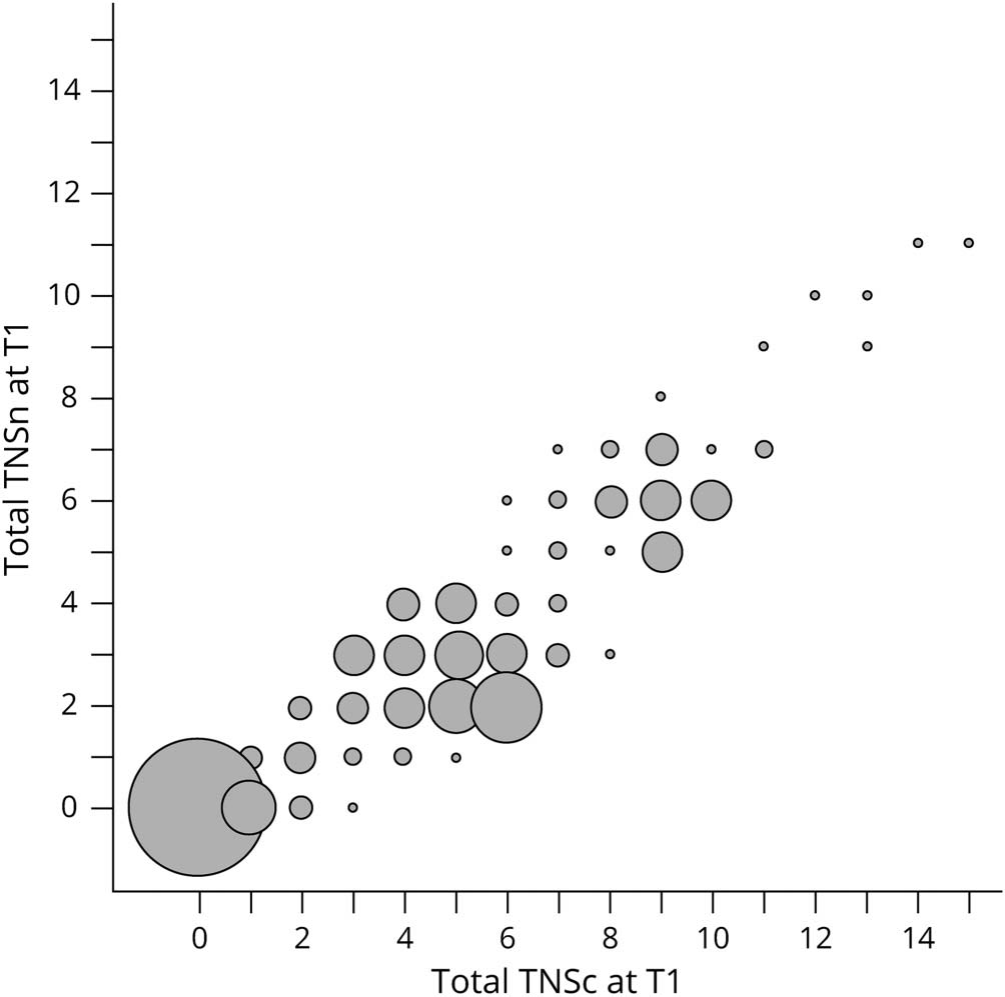
Comparison Between Total Neuropathy Score, Clinical Version (TNSc) and Total Neuropathy Score, Nurse-Assessed Version (TNSn) at T1 The radius of the bubbles is proportional to the absolute frequency. Spearman correlation index = 88.7%.

Neurologic status at the end of observational period was impaired in a substantial proportion of our population: 88% of participants showed any grade neuropathy as assessed via TNSc and 82% according to TNSn. In table 2, end treatment neurologic status is stratified according to chemotherapy regimen administered; there was borderline evidence of difference among the 3 groups in neuropathy severity both according to TNSc and TNSn (table 2). A comparison between TNSc and TNSn values at T1 is shown in figure 1. The Spearman correlation index was 88.7%, indicating that the variability of TNSc is almost fully captured by TNSn.

**Table 2:**
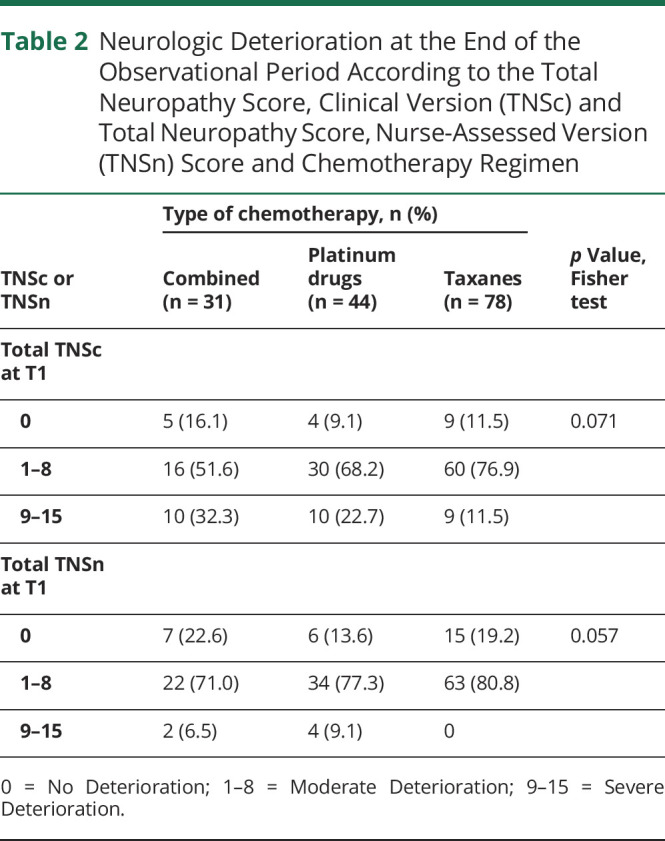
Neurologic Deterioration at the End of the Observational Period According to the Total Neuropathy Score, Clinical Version (TNSc) and Total Neuropathy Score, Nurse-Assessed Version (TNSn) Score and Chemotherapy Regimen

### Relationship Between Physician and Patient-Reported Outcome Measure

**Figure 2:**
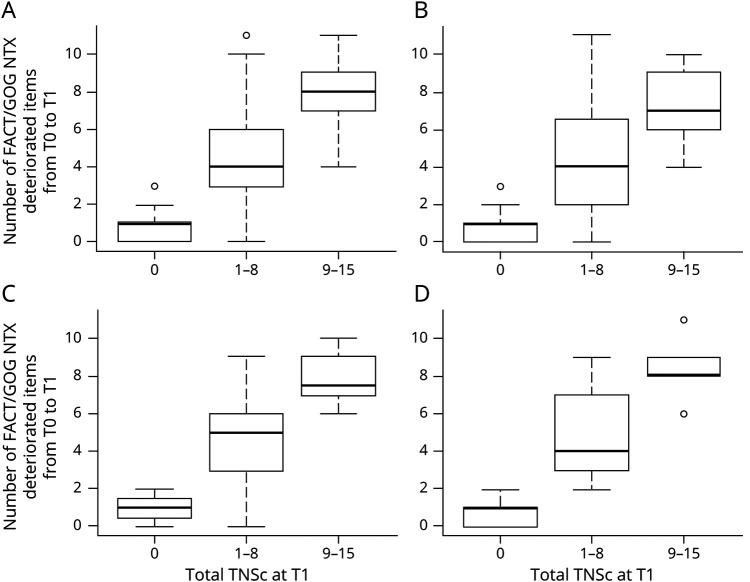
Distribution of Deteriorated Functional Assessment of Cancer Treatment/Gynecologic Oncology Group–Neurotoxicity (FACT/GOG-NTX) Items by Total Neuropathy Score, Clinical Version (TNSc) Category (A) Overall. (B) Patients treated with taxanes. (C) Patients treated with platinum drugs. (D) Patients treated with taxanes + platinum drugs. For all groups, the Kruskal-Wallis test *p* value was <0.001.

**Figure 3:**
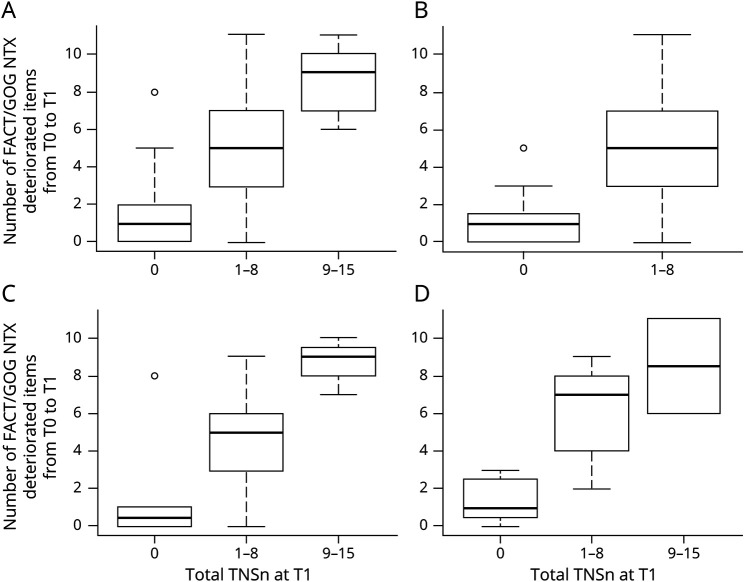
Distribution of Deteriorated Functional Assessment of Cancer Treatment/Gynecologic Oncology Group–Neurotoxicity (FACT/GOG-NTX) Items by Total Neuropathy Score, Nurse-Assessed Version (TNSn) Category (A) Overall. (B) Patients treated with taxanes. (C) Patients treated with platinum drugs. (D) Patients treated with taxanes + platinum drugs. For all groups, the Kruskal-Wallis test *p* value was ≤0.001.

As shown in table 3, we then explored the association between the deterioration of each single FACT/GOG-NTX item and TNSc or TNSn. Table 3 shows data for the overall population; to see data stratified for drug class, see eTable 5 (available at BOARD,^25^ board.unimib.it/research-data/): significance is the same as for the overall population, even when analyzing each class. Again, the triple categorization of TNSc or TNSn was used while deterioration for FACT/GOG-NTX items was intended as a score at end of treatment higher than baseline by at least 1 point. Only the first 4 items of FACT/GOG-NTX (items Ntx1–4) and the last 3 items (Ntx8, Ntx9, and An6) showed a moderate grade of association with TNSc and TNSn, both in the whole population and in each chemotherapy regimen subgroup. A strong association between deterioration of FACT/GOG-NTX taken as a whole and TNSc was observed. As shown in figure 2, the number of deteriorated FACT/GOG-NTX items tended to increase along with TNSc score, overall and in all the chemotherapy regimen subgroups. Again, these findings largely overlap with results regarding the association between FACT/GOG-NTX and TNSn (figure 3).

**Table 3:**
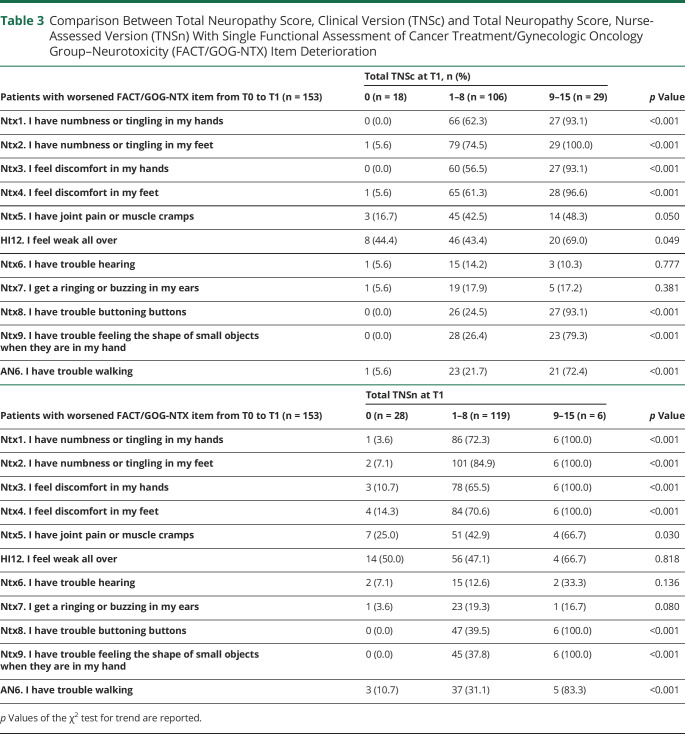
Comparison Between Total Neuropathy Score, Clinical Version (TNSc) and Total Neuropathy Score, Nurse-Assessed Version (TNSn) With Single Functional Assessment of Cancer Treatment/Gynecologic Oncology Group–Neurotoxicity (FACT/GOG-NTX) Item Deterioration

### Minimal Clinically Important Difference

Using an anchor-based approach, considering as clinically important a change in the FACT/GOG-NTX score of at least 3.3 points (0.3 per item) as described by Yost and Eton,^48^ the MCID was calculated for the TNSc and for the TNSn. When using the TNSc the MCID is about 3.7, and it was 2.8 for the TNSn, as shown in table 4.

**Table 4:**
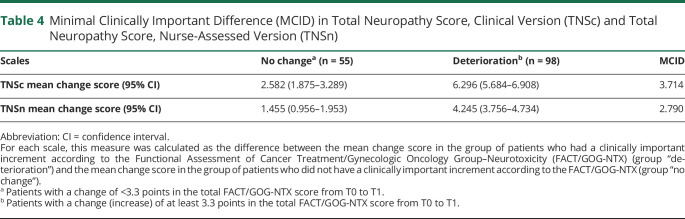
Minimal Clinically Important Difference (MCID) in Total Neuropathy Score, Clinical Version (TNSc) and Total Neuropathy Score, Nurse-Assessed Version (TNSn)

## Discussion

Physicians’ and participants’ perceptions of CIPN, and particularly its effect on quality of life, are different yet complementary.^20,29^ Several groups have addressed CIPN assessment issues^30-39^ considering CRO as well as PRO measures. CROs enable the recognition of CIPN based on the objective treating physician evaluation, whereas PROs provide the individual perception of the patient of her or his condition, a subjective feeling that does not always match clinicians' objective evaluation.^20^ PROs have gained growing attention in recent years for new drug approval and in 2009 the Food and Drug administration delivered specific guidelines to determine what qualifies minimum PRO requisites to be applicable for drug approval.^40^ Between 2011 and 2015, nearly 7% out of 182 new drug approvals had PRO labeling, and more than 75% of PRO labeling was based on primary endpoints. Kluetz et al.^41^ analyzed the issue of incorporating patient experience into the regulatory process in cancer research and recognized the importance and relevance of PROs in benefit/risk assessment in cancer treatment, but also pointed out that a history of poorly defined PRO objectives and methodologies have hampered their usefulness. Therefore, a joint effort of all stakeholders was suggested to improve their reliability and efficacy in CIPN research. On this background, the National Cancer Institute Symptom Management and Health-Related Quality of Life Steering Committee Clinical Trials Planning Meeting was established in 2017 specifically to improve the quality of CIPN clinical research. Dorsey et al.^42^ recently summarized the conclusions of this working group, emphasizing the absence of a validated gold standard and the crucial need of interdisciplinary efforts to unravel CIPN research methodologic issues. The Analgesic, Anesthetic, and Addiction Clinical Trial Translations, Innovations, Opportunities and Networks (ACTTION) Consortium on Clinical Endpoints and Procedures for Peripheral Neuropathy Trials (CONCEPPT) meeting, attended by neurologists, oncologists, pharmacists, clinical trialists, statisticians, and regulatory experts, also analyzed the issue of outcome measures in CIPN clinical trials, giving the recommendation to combine PROs and CROs.^17^

Among PROs, the FACT/GOG-NTX and the European Organization for Research and Treatment of Cancer (EORTC) CIPN20^43^ have gained the most widespread use; among CROs, the TNS or one of its versions such as the TNSc^17,31,38^ showed valid psychometric properties. By contrast, despite being widely used in oncology clinical trials, the NCI-CTCAE raised concerns for appropriateness in CIPN grading and detection^15,23^ and it cannot be suggested as a sole outcome measure to evaluate CIPN. While the TNSc had a significant correlation with the NCI-CTCAE in scoring the severity of CIPN, it showed a higher sensitivity to CIPN changes.^44^ Moreover, the NCI-CTCAE evaluation can overestimate the occurrence of motor neuropathy, possibly because of the presence of confounding factors (e.g., fatigue, depression, cachexia), which might be difficult to rule out without a formal neurologic examination.^23^

Haryani et al.^38^ performed a detailed psychometric evaluation of different available assessment tools in CIPN addressing validity (criterion, construct, discriminant validity), reliability, and practicability; by their extensive investigation, 2 tools emerged as most adequate: a PRO (the FACT/GOG-NTX^45^) and a CRO (the TNS or one of its versions such as the TNSc).^17,31,38^ FACT/GOG-NTX—with respect to other scales such as EORTC CIPN20—has been suggested to be easier to use,^38,46^ and the TNS has been recognized as a fair option for CIPN evaluation by a Delphi survey,^30^ as well as reviews by CIPN experts.^15,33^ Therefore, in our study we focused our attention on these 2 assessment tools.

The original TNS was designed to be performed by trained neuromuscular physicians and included the results of nerve conduction studies (NCS) and a specific quantitative sensory testing (QST) device.^47^ Cavaletti et al.^44,e1-e3^ and others^31,e4-e7^ then studied other versions in which either the QST device was removed or both the QST device and NCS were removed to make the assessment simpler. These have various names, including TNS modified,^e4,e8^ TNS reduced, short-form TNS reduced, or the most popular, TNSc.^44,e3,e4^ Because the TNSc as originally designed required a physician to perform the strength and reflex testing, Cornblath et al. developed the TNSn (unpublished data). This version retains only the original 5 components of the TNS and thus can be done by a trained health care professional. This version has been used extensively in clinical trials, but there is little formal evaluation of it and, in particular, comparison to other CIPN assessments.

Another important concept is emerging in the assessment of CIPN and the effects of treatments (MCID; i.e., the smallest difference in score in the domain of interest that participants perceive as important), either beneficial or harmful, and which would lead the clinician to consider a change in the patient's management.^e9^ The MCID has recently been calculated for FACT/GOG-NTX and EORTC CIPN20,^48,e10^ but this has not been done for any physician-based assessment in CIPN, including any TNS version.

Our data are intended to explore all these issues related to CIPN assessment and to shed light on the best clinimetric approach to this nosographic entity in clinical trials; in the same population of patients with cancer undergoing neurotoxic chemotherapy, we used one of the most recommended PROs, the FACT/GOG-NTX, and the most recommended physician-based outcome scale, TNSc, together. Because of its frequent use in industry and government-sponsored trials, we also employed the NCI-CTCAE.

All 3 scales show that CIPN is a frequent occurrence in this population. We confirmed the internal responsiveness of the 3 outcome measures. However, other studies have shown that the NCI-CTCAE neurotoxicity scales, commonly used in clinical trials, are poorly informative in terms of quality of neurologic impairment.^15^Thus, we would endorse the growing consensus that FACT/GOG-NTX and a form of the TNS be the primary assessment tools in CIPN without NCI-CTCAE.

The original version of the FACT/GOG-NTX is an 11-item questionnaire aimed at exploring positive and negative neuropathy symptoms in CIPN and the consequent functional impairment.^45,e11^ Its clinimetric properties are known^e10^ and the MCID for the FACT/GOG-NTX has been calculated.^48^ Huang et al.^e11^ reexamined the scale with the hypothesis that some of the 11 items might be redundant. They validated a reduced version of the questionnaire based on the first 4 items only (positive and negative neuropathy symptoms in upper and lower limbs). To verify whether other questions might better characterize CIPN, we tested the association between neurologic examination, as assessed by the TNSc, and all single FACT/GOG-NTX items. We confirmed the results obtained by Huang et al.,^e11^ who described significant association between worsening of neurologic status and the first 4 items of the FACT/GOG-NTX; moreover, we verified that there the same association is present with the last 3 items of FACT/GOG-NTX, the ones exploring fine sensory perception and sensory ataxia (i.e., loss of proprioception, relevant to hamper manipulation and balance): Ntx8 “having trouble buttoning buttons,” Ntx9 “having trouble feeling the shape of small objects,” and An6 “having trouble walking.” No association was observed with the remaining items. We conclude that the complete 11-item FACT/GOG-NTX is not needed, but rather a 7-item reduced version is the most informative.

We also tested a shorter version of the TNSc—the TNSn—that could be easily and rapidly employed in any oncologic center by a trained health care professional. The TNSn had significant responsiveness and showed the same association with FACT/GOG-NTX items as observed with the full TNSc.

As a final analysis aimed at providing information regarding a widely used physician-based outcome measure in CIPN, we defined the MCID for both the TNSc and TNSn scales. This provides cutoff values for a relevant change that could drive clinical practice and allow better definition of relevant endpoints in CIPN clinical trials. In order to perform this analysis, we used the MCID for the FACT/GOG-NTX^48^ as a reference. As expected, the MCID was higher using the TNSc if compared with the TNSn (approximatively 3.7 vs 2.8), reflecting the different value range of the 2 scales (0–28 vs 0–20, respectively) maintaining indeed a similar “relative” MCID (3.7/28 = 1.3% vs 2.8/20 = 1.4%).

This study provides Class III evidence that for participants receiving neurotoxic chemotherapy, a patient-completed questionnaire and nurse-assessed scale moderately correlate with a physician-assessed neuropathy scale. Our study adds important and new information to an evidence-based selection of the most appropriate tools in the assessment of CIPN. We show that both FACT/GOG-NTX and TNSc can measure neuropathy in a real-life population of participants with cancer recruited in a multisite, international study. These results were consistent among different drugs and drug combinations, suggesting they could be used across multiple cancer treatment regimens. Our data support the use of a shorter FACT/GOG-NTX scale, indicating that a 7-item scale would be the most suitable option to capture sensory ataxia and its effect on daily life activities. Lastly, we defined the MCID for the TNSc and demonstrated that the TNSn can be considered a reliable alternative if a formal neurologic examination by physicians or specifically trained nurses are not possible in a specific center. The selected simple set of measures for CIPN are clinimetrically valid, do not need complex training, and can be used easily in trials anywhere.

## Glossary

CI: confidence interval
CIPN: chemotherapy-induced peripheral neurotoxicity
CRO: clinician-reported outcome
EORTC: European Organization for Research and Treatment of Cancer
FACT/GOG-NTX: Functional Assessment of Cancer Treatment/Gynecologic Oncology Group–Neurotoxicity
MCID: minimal clinically important difference
NCI-CTCAE: National Cancer Institute Common Terminology Criteria for Adverse Events
NCS: nerve conduction studies
PRO: patient-reported outcome
QST: quantitative sensory testing
TNS: Total Neuropathy Score
TNSc: Total Neuropathy Score, clinical version
TNSn: Total Neuropathy Score, nurse-assessed version

## References

[1] Staff NP, Grisold A, Grisold W, Windebank AJ. Chemotherapy-induced peripheral neuropathy: a current review. Ann Neurol. 2017;81(6):772-781. 28486769 10.1002/ana.24951 PMC5656281

[2] Shah A, Hoffman EM, Mauermann ML, et al. Incidence and disease burden of chemotherapy-induced peripheral neuropathy in a population-based cohort. J Neurol Neurosurg Psychiatry. 2018;89(6):636-641. 29439162 10.1136/jnnp-2017-317215 PMC5970026

[3] Cavaletti G, Marmiroli P. Chemotherapy-induced peripheral neurotoxicity. Curr Opin Neurol. 2015;28(5):500-507. 26197027 10.1097/WCO.0000000000000234

[4] Cavaletti G, Alberti P, Argyriou AA, et al. Chemotherapy-induced peripheral neurotoxicity: a multifaceted, still unsolved issue. J Peripher Nerv Syst. 2019;24(suppl 2):S6-S12. 31647155 10.1111/jns.12337

[5] Briani C, Argyriou AA, Izquierdo C, et al. Long-term course of oxaliplatin-induced polyneuropathy: a prospective 2-year follow-up study. J Peripher Nerv Syst. 2014;19(4):299-306. 25582667 10.1111/jns.12097

[6] Velasco R, Alberti P, Bruna J, Psimaras D, Argyriou AA. Bortezomib and other proteosome inhibitors-induced peripheral neurotoxicity: from pathogenesis to treatment. J Peripher Nerv Syst. 2019;24(suppl 2):S52-S62. 31647153 10.1111/jns.12338

[7] Tamburin S, Park SB, Alberti P, Demichelis C, Schenone A, Argyriou AA. Taxane and epothilone-induced peripheral neurotoxicity: from pathogenesis to treatment. J Peripher Nerv Syst. 2019;24(suppl 2):S40-S51. 31647157 10.1111/jns.12336

[8] Islam B, Lustberg M, Staff NP, Kolb N, Alberti P, Argyriou AA. Vinca alkaloids, thalidomide and eribulin-induced peripheral neurotoxicity: from pathogenesis to treatment. J Peripher Nerv Syst. 2019;24(suppl 2):S63-S73. 31647152 10.1111/jns.12334

[9] Staff NP, Cavaletti G, Islam B, Lustberg M, Psimaras D, Tamburin S. Platinum-induced peripheral neurotoxicity: from pathogenesis to treatment. J Peripher Nerv Syst. 2019;24(suppl 2):S26-S39. 31647151 10.1111/jns.12335 PMC6818741

[10] Bjornard KL, Gilchrist LS, Inaba H, et al. Peripheral neuropathy in children and adolescents treated for cancer. Lancet Child Adolesc Health. 2018;2(10):744-754. 30236383 10.1016/S2352-4642(18)30236-0 PMC6287277

[11] Selvy M, Pereira B, Kerckhove N, et al. Long-term prevalence of sensory chemotherapy-induced peripheral neuropathy for 5 years after adjuvant FOLFOX chemotherapy to treat colorectal cancer: a multicenter cross-sectional study. J Clin Med. 2020;9(8):2400. 32727095 10.3390/jcm9082400 PMC7465246

[12] Cavaletti G, Alberti P, Marmiroli P. Chemotherapy-induced peripheral neurotoxicity in cancer survivors: an underdiagnosed clinical entity? Am Soc Clin Oncol Ed Book. 2015:e553-e560. 10.14694/EdBook_AM.2015.35.e553 25993222

[13] Loprinzi CL, Lacchetti C, Bleeker J, et al. Prevention and management of chemotherapy-induced peripheral neuropathy in survivors of adult cancers: ASCO guideline update. J Clin Oncol. 2020;38(28):3325-3348. 32663120 10.1200/JCO.20.01399

[14] Liew WK, Pacak CA, Visyak N, Darras BT, Bousvaros A, Kang PB. Longitudinal patterns of thalidomide neuropathy in children and adolescents. J Pediatr. 2016;178:227-232. 27567409 10.1016/j.jpeds.2016.07.040

[15] Cavaletti G, Frigeni B, Lanzani F, et al. Chemotherapy-induced peripheral neurotoxicity assessment: a critical revision of the currently available tools. Eur J Cancer. 2010;46(3):479-494. 20045310 10.1016/j.ejca.2009.12.008

[16] Gewandter JS, Gibbons CH, Campagnolo M, et al. Clinician-rated measures for distal symmetrical axonal polyneuropathy: ACTTION systematic review. Neurology. 2019;93(8):346-360. 31320471 10.1212/WNL.0000000000007974

[17] Gewandter JS, Brell J, Cavaletti G, et al. Trial designs for chemotherapy-induced peripheral neuropathy prevention: ACTTION recommendations. Neurology. 2018;91(9):403-413. 30054438 10.1212/WNL.0000000000006083 PMC6133627

[18] Gewandter JS, Burke L, Cavaletti G, et al. Content validity of symptom-based measures for diabetic, chemotherapy, and HIV peripheral neuropathy. Muscle Nerve. 2017;55(3):366-372. 27447116 10.1002/mus.25264 PMC5528159

[19] Gewandter JS, Freeman R, Kitt RA, et al. Chemotherapy-induced peripheral neuropathy clinical trials: review and recommendations. Neurology. 2017;89(8):859-869. 28747442 10.1212/WNL.0000000000004272 PMC10681068

[20] Alberti P, Rossi E, Cornblath DR, et al. Physician-assessed and patient-reported outcome measures in chemotherapy-induced sensory peripheral neurotoxicity: two sides of the same coin. Ann Oncol. 2014;25(1):257-264. 24256846 10.1093/annonc/mdt409 PMC3868322

[21] Griffith KA, Dorsey SG, Renn CL, et al. Correspondence between neurophysiological and clinical measurements of chemotherapy-induced peripheral neuropathy: secondary analysis of data from the CI-PeriNomS study. J Peripher Nerv Syst. 2014;19(2):127-135. 24814100 10.1111/jns5.12064 PMC4175057

[22] Cavaletti G, Cornblath DR, Merkies IS, et al. The chemotherapy-induced peripheral neuropathy outcome measures standardization study: from consensus to the first validity and reliability findings. Ann Oncol. 2013;24(2):454-462. 22910842 10.1093/annonc/mds329 PMC3551481

[23] Frigeni B, Piatti M, Lanzani F, et al. Chemotherapy-induced peripheral neurotoxicity can be misdiagnosed by the National Cancer Institute Common Toxicity scale. J Peripher Nerv Syst. 2011;16(3):228-236. 22003937 10.1111/j.1529-8027.2011.00351.x

[24] Frigeni B, Piatti M, Lanzani F, et al. The total neuropathy score is more reliable than the national cancer institute common toxicity scale to assess stable chemotherapy-induced peripheral neurotoxicity. J Peripher Nerv Syst. 2011;16:S42-S43. 10.1111/j.1529-8027.2011.00351.x 22003937

[25] Supplementary tables/figures and references to A Prospective Evaluation of Health Care Provider and Patient Assessments in Chemotherapy Induced Peripheral Neurotoxicity. Bicocca Open Archive Research Data. Accessed April 9, 2021. 10.17632/283rk2x353.2. PMC10365895 34078718

[26] Husted JA, Cook RJ, Farewell VT, Gladman DD. Methods for assessing responsiveness: a critical review and recommendations. J Clin Epidemiol. 2000;53(5):459-468. 10812317 10.1016/s0895-4356(99)00206-1

[27] Argyriou AA, Cavaletti G, Antonacopoulou A, et al. Voltage-gated sodium channel polymorphisms play a pivotal role in the development of oxaliplatin-induced peripheral neurotoxicity: results from a prospective multicenter study. Cancer. 2013;119(19):3570-3577. 23821303 10.1002/cncr.28234

[28] Revicki D, Hays RD, Cella D, Sloan J. Recommended methods for determining responsiveness and minimally important differences for patient-reported outcomes. J Clin Epidemiol. 2008;61(2):102-109. 18177782 10.1016/j.jclinepi.2007.03.012

[29] Cavaletti G, Cornblath DR, Merkies ISJ, et al. Patients' and physicians' interpretation of chemotherapy-induced peripheral neurotoxicity. J Peripher Nerv Syst. 2019;24(1):111-119. 30672664 10.1111/jns.12306

[30] McCrary JM, Goldstein D, Boyle F, et al. Optimal clinical assessment strategies for chemotherapy-induced peripheral neuropathy (CIPN): a systematic review and Delphi survey. Support Care Cancer. 2017;25(11):3485-3493. 28589310 10.1007/s00520-017-3772-y

[31] Smith EML, Knoerl R, Yang JJ, Kanzawa-Lee G, Lee D, Bridges CM. In search of a gold standard patient-reported outcome measure for use in chemotherapy- induced peripheral neuropathy clinical trials. Cancer Control. 2018;25(1):1073274818756608. 29480026 10.1177/1073274818756608 PMC5925747

[32] Ibrahim EY, Ehrlich BE. Prevention of chemotherapy-induced peripheral neuropathy: a review of recent findings. Crit Rev Oncol Hematol. 2020;145:102831. 31783290 10.1016/j.critrevonc.2019.102831 PMC6982645

[33] Park SB, Alberti P, Kolb NA, Gewandter JS, Schenone A, Argyriou AA. Overview and critical revision of clinical assessment tools in chemotherapy-induced peripheral neurotoxicity. J Peripher Nerv Syst 2019;24(suppl 2):S13-S25. 31647154 10.1111/jns.12333

[34] Wang M, Cheng HL, Lopez V, Sundar R, Yorke J, Molassiotis A. Redefining chemotherapy-induced peripheral neuropathy through symptom cluster analysis and patient-reported outcome data over time. BMC Cancer. 2019;19(1):1151. 31775665 10.1186/s12885-019-6352-3 PMC6882224

[35] Kanda K, Fujimoto K, Mochizuki R, Ishida K, Lee B. Development and validation of the comprehensive assessment scale for chemotherapy-induced peripheral neuropathy in survivors of cancer. BMC Cancer. 2019;19(1):904. 31506070 10.1186/s12885-019-6113-3 PMC6734590

[36] Yoshida Y, Satoh A, Yamada T, et al. The relationship between evaluation methods for chemotherapy-induced peripheral neuropathy. Sci Rep. 2019;9(1):20361. 31889149 10.1038/s41598-019-56969-9 PMC6937307

[37] Tan AC, McCrary JM, Park SB, Trinh T, Goldstein D. Chemotherapy-induced peripheral neuropathy-patient-reported outcomes compared with NCI-CTCAE grade. Support Care Cancer. 2019;27(12):4771-4777. 30972648 10.1007/s00520-019-04781-6

[38] Haryani H, Fetzer SJ, Wu CL, Hsu YY. Chemotherapy-induced peripheral neuropathy assessment tools: a systematic review. Oncol Nurs Forum. 2017;44(3):E111-E123. 28635977 10.1188/17.ONF.E111-E123

[39] Mendoza TR, Williams LA, Shi Q, et al. The Treatment-induced Neuropathy Assessment Scale (TNAS): a psychometric update following qualitative enrichment. J Patient Rep Outcomes. 2020;4(1):15. 32076879 10.1186/s41687-020-0180-8 PMC7031452

[40] Guidance for Industry Patient-Reported Outcome Measures: Use in Medical Product Development to Support Labeling Claims. Food and Drug Administration. Accessed April 9, 2021. fda.gov/media/77832/download

[41] Kluetz PG, O'Connor DJ, Soltys K. Incorporating the patient experience into regulatory decision making in the USA, Europe, and Canada. Lancet Oncol. 2018;19(5):e267-e274. 29726391 10.1016/S1470-2045(18)30097-4

[42] Dorsey SG, Kleckner IR, Barton D, et al. NCI Clinical Trials Planning Meeting for prevention and treatment of chemotherapy-induced peripheral neuropathy. J Natl Cancer Inst 2019;111(6):531-537. 30715378 10.1093/jnci/djz011 PMC7962883

[43] Molassiotis A, Cheng HL, Lopez V, et al. Are we mis-estimating chemotherapy-induced peripheral neuropathy? Analysis of assessment methodologies from a prospective, multinational, longitudinal cohort study of patients receiving neurotoxic chemotherapy. BMC Cancer. 2019;19(1):132. 30736741 10.1186/s12885-019-5302-4 PMC6368751

[44] Cavaletti G, Frigeni B, Lanzani F, et al. The Total Neuropathy Score as an assessment tool for grading the course of chemotherapy-induced peripheral neurotoxicity: comparison with the National Cancer Institute-Common Toxicity Scale. J Peripher Nerv Syst. 2007;12(3):210-215. 17868248 10.1111/j.1529-8027.2007.00141.x

[45] Calhoun EA, Welshman EE, Chang CH, et al. Psychometric evaluation of the Functional Assessment of Cancer Therapy/Gynecologic Oncology Group-Neurotoxicity (Fact/GOG-Ntx) questionnaire for patients receiving systemic chemotherapy. Int J Gynecol Cancer. 2003;13(6):741-748. 14675309 10.1111/j.1525-1438.2003.13603.x

[46] McCrary JM, Goldstein D, Trinh T, et al. Optimizing clinical screening for chemotherapy-induced peripheral neuropathy. J Pain Symptom Manage. 2019;58(6):1023-1032. 31374367 10.1016/j.jpainsymman.2019.07.021

[47] Cornblath DR, Chaudhry V, Carter K, et al. Total neuropathy score: validation and reliability study. Neurology. 1999;53(8):1660-1664. 10563609 10.1212/wnl.53.8.1660

[48] Yost KJ, Eton DT. Combining distribution- and anchor-based approaches to determine minimally important differences: the FACIT experience. Eval Health Prof. 2005;28(2):172-191. 15851772 10.1177/0163278705275340

